# Case series of acrodermatitis chronica atrophicans in the United States with absent or remote European travel

**DOI:** 10.1016/j.jdcr.2025.12.037

**Published:** 2025-12-29

**Authors:** Julia Giroski, Nicholas Flint, Andrew Desrosiers, Oksana Bailiff, Allen Strickler, Tammie Ferringer

**Affiliations:** a School of Medicine, Geisinger, Scranton, Pennsylvania; b Department of Dermatology, Geisinger, Danville, Pennsylvania; c Department of Laboratory Medicine, Geisinger, Danville, Pennsylvania

**Keywords:** acrodermatitis chronica atrophicans, Borrelia, cutaneous atrophy, Lyme disease, tick-borne illness

## Introduction

Acrodermatitis chronica atrophicans (ACA) is a chronic late-stage cutaneous manifestation of Lyme disease that is rarely reported in the United States and is endemic to Europe.^1^^,^^2^ We present the only known reported case of ACA in the United States where the patient has no prior history of European or other international travel. All other cases of ACA in the United States have been reported in European immigrants or patients who have traveled to Europe.^3^ Notably, ACA manifests in about 10% of Lyme disease cases reported in Europe.^2^ Lyme disease is a multisystem disorder caused by the spirochete *Borrelia burgdorferi* sensu lato which has been subdivided into 3 genospecies: *Borrelia burgdorferi* sensu stricto (usually referred to as *Borrelia burgdorferi*), *Borrelia afzelii*, and *Borrelia garinii*. Manifestations include neurological, cardiovascular, joint, and cutaneous symptoms.^4^ ACA is most commonly caused by the genospecies *Borrelia afzelii,* and less frequently *Borrelia garinii.*^2^^,^^4^ Clinically, ACA manifests as a bluish-red discoloration predominantly on acral sites, particularly on the lower extremities. Over time, lesions may become atrophic or sclerotic. Neuropathic pain, arthropathy, and osseous changes may also be observed.^1^ ACA lesions often develop years after initial spirochete infection and typically persist without spontaneous resolution unless treated.^1^

Here, we present a 71-year-old woman with clinical and histological findings consistent with ACA and no relevant travel history. Additionally, we present 2 cases of ACA in the United States where the patients’ history of European travel was remote.

## Discussion of cases

### Case 1

A 71-year-old Pennsylvanian woman presented with a 4-year history of violaceous discoloration, initially localized to the right foot, subsequently progressing to bilateral involvement. These cutaneous changes were accompanied by patient-reported, progressive foot enlargement from a size 7 to a size 9 and marked tenderness to palpation. The patient also reported multiple tick exposures over the preceding 10-15 years and a history of lower extremity rashes in 2016-2017, treated empirically with cephalexin. She denied ever observing the characteristic erythema chronicum migrans rash. Lyme serologies (immunoglobulin M [IgM] and immunoglobulin G [IgG]) were positive on 3 occasions—2011, 2012, and 2015. She had no history of travel outside of the United States.

**Fig 1:**
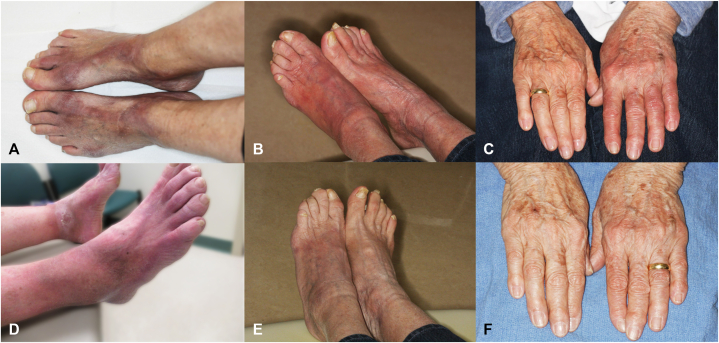
Acrodermatitis chronica atrophicans. **A,** Case 1 dorsal feet at presentation. **B,** Case 2 dorsolateral right foot, medial left foot at presentation. **C** and **D,** Case 3 dorsal hands and feet before treatment. **E** and **F,** Case 3 dorsal hands and feet after treatment with doxycycline monohydrate 100 mg twice daily for 28 days.

Physical examination demonstrated symmetric red brown to violaceous sclerotic patches and plaques on the bilateral dorsal feet, predominantly overlying bony prominences (Fig 1, *A*). The lesions were notably tender to palpation. The remainder of the examination was unremarkable.

**Fig 2:**
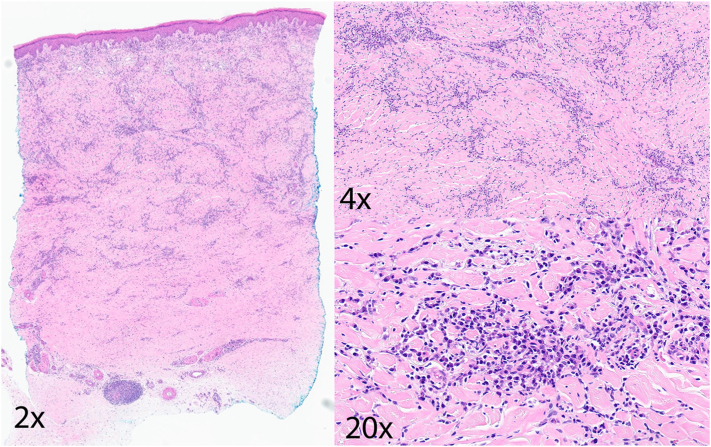
Acrodermatitis chronica atrophicans. Case 1. Hematoxylin and eosin-stained sections revealed a relatively unremarkable epidermis. The dermis showed a lymphoplasmacytic infiltrate with background sclerotic collagen.

A punch biopsy obtained from the right dorsal foot revealed sclerosing dermatosis with a plasmacytic infiltrate, supportive of ACA (Fig 2).^5^ Lyme disease antibody screen with reflex to confirmatory testing demonstrated IgG and IgM positivity; however, the interpretation was limited given the patient’s prior documented seropositivity. Notably, IgG and IgM antibodies may persist for years following eradication of the infection, reducing the specificity of these findings for active disease.^6^ The patient was treated with doxycycline monohydrate 100 mg twice daily for 28 days. At 1-month follow-up, she reported significant reduction in tenderness, which had been a primary factor impacting her quality of life.

### Case 2

An 80-year-old woman presented with a 2 to 3–year history of progressively worsening, painful discoloration of the bilateral feet. The patient described the affected areas as burning and hypersensitive, with allodynia severe enough that even light pressure from bed covers elicited discomfort. Associated symptoms included left lower extremity edema, which impaired her ability to wear footwear and engage in physical activity. She reported having to cut the tongues out of her shoes to accommodate the swelling. Her travel history included multiple trips to Europe, most recently in the winter of 2011, 8 years prior to symptom onset. She recalled a single episode of erythema chronica migrans rash prior to that trip, attributed to a tick bite in the United States, which was treated with doxycycline. She did not report any tick exposures, erythema chronica migrans, or symptoms suggestive of Lyme disease during or following international travel. In 2016, she was diagnosed with Lyme disease, confirmed serologically and treated with amoxicillin and doxycycline. Her medical history was also notable for monoclonal gammopathy of undetermined significance.

The patient had previously been evaluated by multiple specialists without definitive diagnosis for her symptoms. Circulatory testing was unremarkable. In July 2020, approximately 6 months prior to presentation to the dermatology clinic, she was evaluated by a rheumatologist who considered erythromelalgia as a possible etiology and referred her for dermatological evaluation and skin biopsy.

Physical examination revealed blanchable erythematous to violaceous indurated plaques on the bilateral dorsal feet and ankles (Fig 1, *B*).

A punch biopsy of the right medial ankle revealed dermal fibrosis and diffuse lymphohistiocytic infiltrate with plasma cells. Lyme disease antibody screen was positive, and confirmatory western blot demonstrated reactivity in all 10 IgG bands with no IgM band reactivity. However, the clinical significance of these findings is uncertain given the patient’s prior diagnosis of Lyme disease and positive serological results.

Based on the clinical and histopathological findings consistent with ACA, the patient was initiated on doxycycline monohydrate 100 mg twice daily. After 1 month of therapy, there was a marked reduction in the erythema and pain, facilitating improved footwear tolerance. She was hesitant to stop the doxycycline due to the significant symptom improvement. At 3-month follow-up, with ongoing treatment, she reported that her feet felt the best they had in 2-3 years. The burning sensation and pain continued to improve, enabling resumption of physical activity. However, upon cessation of doxycycline, the patient experienced recurrence of pain, necessitating reinstitution of therapy with a gradual dose reduction to 50 mg daily based on symptom severity. Despite clinical improvement in discomfort, the cutaneous discoloration demonstrated fluctuating persistence.

### Case 3

A 75-year-old female presented with a 6-month history of blotchy discoloration involving the left foot and ankle, with similar involvement in the left hand and less pronounced involvement of the right foot. The initial symptoms reportedly began 13 months prior following a left ankle sprain associated with localized swelling. Multiple imaging modalities were unremarkable, and the swelling subsequently resolved. Approximately 6 months post injury, she developed progressively worsening blotchy erythematous to violaceous discoloration of the left foot and ankle, which intensified throughout the day. These cutaneous changes were accompanied by ankle pain and swelling and polyarticular swelling involving bilateral hand joints, all of which worsened as the day progressed and were exacerbated by heat and physical activity. A short course of methylprednisolone yielded partial improvement of swelling and rash, although symptoms did not completely resolve. The patient denied any prior diagnosis of Lyme disease, but she resided in a heavily wooded region of Pennsylvania endemic for tick exposure. Her travel history was significant only for a trip to France in 1998, 19 years prior to presentation, with no recollection of tick bites or erythema chronica migrans rash during or following that period.

Physical examination revealed diffuse erythematous and violaceous discoloration and induration of the dorsal feet, more pronounced on the left side. Similar findings were observed in the left hand, where swelling resulted in inability to wear her wedding band (Fig 1, *C* and *D*).

Biopsy of the left ankle revealed fibrosis with numerous plasma cells. Lyme disease antibody testing was positive, and confirmatory western blot demonstrated a combined IgG and IgM antibody value more than 12.4, with reactivity in 5 of 10 IgG bands and 2 of 3 IgM bands, consistent with a confirmed Lyme disease infection.

The patient received doxycycline monohydrate 100 mg twice daily for 28 days. At 2-month follow-up, clinical improvement was noted, including marked reduction in discoloration of the left foot and hands and decreased joint swelling in the hands and left ankle (Fig 1, *E* and *F*), enabling the patient to resume wearing her wedding band on the left hand.

## Discussion

These cases document the presence of ACA within the United States, including the only published case of ACA confirmed in a patient born in the United States with no history of international travel. All 3 patients exhibited clinical, histopathological, and serological evidence to support their diagnosis of ACA. Notably, case 1 involved a patient without any reported travel history outside of the United States. Cases 2 and 3 reported no recollection of tick exposure or erythema chronica migrans during or following their respective travels to Europe. Collectively, these cases suggest that ACA is possible from Lyme infection contracted domestically, even in the absence of European travel, reinforcing the importance of health care practitioner awareness of this entity and presentation.

Review of the literature revealed a scarcity of ACA cases reported in the United States. Lavoie et al (1986) described a 58-year-old female California native with ACA, although her travel history was undocumented.^7^ Kaufman et al (1989) reported 2 cases of ACA in a 24-year-old woman and an 18-year-old woman, both native to Long Island, New York. Similarly, their travel histories were omitted.^8^ Edwards et al (1992) documented a case of ACA in a 68-year-old New York native with a history of travel to Europe 15 years prior.^9^ Details of these cases and additional North American ACA cases are outlined in Table I. In each case, ACA was observed in patients who had been born in Europe, had a confirmed history of travel to Europe, or had not disclosed their birthplace or travel history. This highlights the distinctiveness of Case 1, in which the patient explicitly confirmed that she had never traveled outside the United States. Consistent with our cohort, the ACA cases summarized in Table I predominantly involved the lower extremities and were reported in female patients. Treatment regimens primarily involved doxycycline or penicillin, which resulted in improvement in systemic symptoms, local discomfort, and erythema, although atrophic and sclerotic sequelae frequently persisted. Notably, 3 patients experienced relapse upon cessation of antibiotic therapy, necessitating prolonged or repeated treatment courses extending up to 3 years.^10^

**Table I:** North American cases of acrodermatitis chronica atrophicans

Cases of ACA in North America										
Article	Age	Sex	Birthplace	Travel history	Location of ACA	Additional symptoms	Year of presentation	Treatment	Outcome	Confirmation of infection
DiCaudo et al^3^	59	F	Norway	N/A	Feet, legs	Paresthesia, arthritis of spine and hips	1943	N/A	N/A	N/A
DiCaudo et al^3^	62	M	Latvia	N/A	Hand, elbow	N/A	1961	Penicillin, dose unspecified	Noted clinical response	N/A
DiCaudo et al^3^	57	M	Russia	N/A	Arms, feet	Paresthesia	1937	N/A	N/A	N/A
DiCaudo et al^3^	39	F	Poland	N/A	Leg	N/A	1938	N/A	N/A	N/A
DiCaudo et al^3^	56	F	Germany	N/A	Legs	Pruritus	1927	N/A	N/A	N/A
DiCaudo et al^3^	62	F	N/A	N/A	Arms, legs	Paresthesia, arthritis of ankles and feet	1932	N/A	N/A	N/A
DiCaudo et al^3^	81	F	United States	Traveled to Italy, France, and Japan in the 1970s and 1980s	Hand, knees, legs, feet	Paresthesia, fatigue	N/A	Doxycycline monohydrate 100 mg twice daily for 4 wk	Complete resolution of lesions and symptoms 1 mo after completing antibiotic therapy	N/A
Scott, JD^10^	72	M	Canada	N/A	Dorsal knee	Cyclical fever, flu-like symptoms, encephalopathic symptoms	N/A	Ongoing unspecified antibiotic treatment	Resolution of encephalopathic symptoms with persistence of all other symptoms	*B burgdorferi* sensu stricto confirmed by PCR and DNA sequencing
Scott, JD^10^	71	M	Hungary	N/A	Hands	Arthritis	N/A	Doxycycline 100 mg twice a day for 2 mo, 9 subsequent rounds of treatment	Symptoms improved while on antibiotic therapy and were exacerbated in between courses of antibiotics	N/A
Scott, JD^10^	14	M	Ontario	No out of province travel	Calf, foot	Swelling and osteomyelitis of right foot	N/A	Doxycycline 100 mg twice a day, 2-mo sequelae totaling 3 y	Symptoms resolved after 3 y of treatment	*B burgdorferi* sensu stricto confirmed by Western Blot
Scott, JD^10^	64	M	Canada	N/A	Legs, arms	Paresthesia, pruritus, fatigue, swelling, arthritis, muscle aches	N/A	Disulfiram 125 mg, once every third day for 30 d	Symptoms gradually improved with residual fatigue	N/A
Cardenas-de la Garza et al^11^	37	F	Northern Mexico	Never traveled outside of Northern Mexico	Feet, lower legs	None	N/A	Doxycycline (dose unspecified) for 21 d	Partial improvement of rash	*B afzelli* confirmed by ELISA, immunoblot assay, and PCR
Lavoie et al^7^	58	F	United States	N/A	Thigh, feet, elbows, prepatellar regions	Ascending pain and hyperesthesia from ankles to nuchal region, bilateral Bell’s palsies	1976	Short course of oral prednisone, 12 wk of oral penicillin	Resolution of Bell’s palsies, hyperesthesia, and erythema with minimal improvement of atrophic tissues	N/A
Edwards et al^9^	68	M	United States	Traveled to Great Britain 15 y prior to onset of symptoms	Extensor surfaces of the limbs and joints	Mild hearing loss	N/A	Penicillin for 6 wk	No improvement of symptoms	N/A
Kaufman et al^8^	24	F	United States	N/A	Arm, feet	Paresthesia, stiffness in hands and feet, bilateral hip arthralgias, digital blanching and cyanosis	N/A	Patient declined therapy	Unavailable	N/A
Kaufman et al^8^	18	F	United Stated	N/A	Forearm	Arthralgias, fatigue	N/A	Amoxicillin 500 mg, 3 times a day for 3 wk, and probenecid 500 mg 3 times a day for 3 wk	Arthralgias and fatigue resolved 9 mo after therapy, with improvement of rash	N/A
Case 1	71	F	United States	Never traveled outside of the United States	Foot	Bilateral foot enlargement, pain to palpation	2024	Doxycycline monohydrate 100 mg twice daily for 28 d followed by tacrolimus ointment	Rash improved; tenderness greatly decreased	N/A
Case 2	80	F	United States	Traveled to Europe multiple times, most recently in winter 2011	Feet	Severe paresthesia, swelling of left leg	2021	Doxycycline monohydrate 100 mg twice daily for over 2 y, then 100 mg daily for 1 y, then 50 mg daily	Paresthesia and swelling resolved, with some residual discoloration of the feet	N/A
Case 3	75	F	United States	Traveled to France in 1998	Foot, ankle, hand	Ankle pain and swelling, swelling of the joints of both hands	2017	Doxycycline monohydrate 100 mg twice a day for 30 d	Discoloration improved, swelling decreased	N/A

*ACA*, Acrodermatitis chronica atrophicans; *B burgdorferi*, *Borrelia burgdorferi*; *ELISA*, enzyme-linked immunosorbent assay; *F*, female; *M*, male; *N/A*, not available; *PCR*, polymerase chain reaction.

*Borrelia afzelii*, the principal etiologic agent of ACA, is not considered endemic to the United States. Given ACA’s protracted latency after initial infection, some North American cases may reflect prior exposure to *Borrelia afzelii* in endemic regions via international travel. However, patient 1 explicitly denied any such travel. The global expansion of ticks and associated tick-borne pathogens has the potential to introduce novel ticks and pathogens to previously unaffected regions. *Ixodes ricinus*, the primary tick vector of *Borrelia afzelii*, has recently been reported in the United States due to increasing global travel and trade.^12^

The majority of Lyme disease cases in the United States are caused by *Borrelia burgdorferi* sensu stricto, transmitted by *Ixodes scapularis*, a genospecies rarely implicated in ACA.^4^^,^^13^ However, 2 cases of ACA attributable to *Borrelia burgdorferi* sensu stricto have been reported in Canada,^10^ and *Borrelia burgdorferi* sensu stricto was identified in 6% of skin biopsies from a cohort of 693 Slovenian patients diagnosed with ACA, providing further evidence of the genospecies’ etiological role in the pathogenesis of ACA.^13^ Furthermore, a 2016 study comparing the strains of *Borrelia burgdorferi* sensu stricto in Central Europe with those in the United States demonstrated that despite phylogenetic similarities, the European strains manifest clinical phenotypes more similar to *Borrelia afzelii*.^14^

The specific genotypes underlying infection in our patients remain undetermined. It is plausible that infection resulted from nonendemic genotypes introduced through travel. Alternatively, ACA in the United States may represent an under-recognized or very rare clinical phenotype of *Borrelia burgdorferi* sensu stricto or a result of genetic convergence to the ACA phenotype in *Borrelia burgdorferi* sensu stricto over time.

Whether attributable to infection acquired during travel, vector and pathogen importation, or underappreciated phenotype or genetic plasticity of endemic *Borrelia* species, ACA exists in the North American context. These findings emphasize the need for increased clinician awareness to facilitate a timely diagnosis and appropriate management of this late-stage Lyme disease manifestation.

## Conflicts of interest

None disclosed.

## References

[1] Leslie T.A., Levell N.J., Cutler S.J. (1994). Acrodermatitis chronica atrophicans: a case report and review of the literature. Br J Dermatol.

[2] Smetanick M.T., Zellis S.L., Ermolovich T. (2010). Acrodermatitis chronica atrophicans: a case report and review of the literature. Cutis.

[3] DiCaudo D.J., Su W.P.D., Marshall W.F. (1994). Acrodermatitis chronica atrophicans in the United States: clinical and histopathological features of six cases. Cutis.

[4] Steere A.C., Strle F., Wormser G.P. (2016). Lyme borreliosis. Nat Rev Dis Primers.

[5] Lenormand C., Jaulhac B., Debarbieux S. (2016). Expanding the clinicopathological spectrum of late cutaneous Lyme borreliosis (acrodermatitis chronica atrophicans [ACA]): a prospective study of 20 culture- and/or polymerase chain reaction (PCR)-documented cases. J Am Acad Dermatol.

[6] Lantos P.M., Rumbaugh J., Bockenstedt L.K. (2021). Clinical Practice guidelines by the Infectious Diseases Society of America (IDSA), American Academy of Neurology (AAN), and American College of Rheumatology (ACR): 2020 guidelines for the prevention, diagnosis, and treatment of lyme disease. Arthritis Rheumatol.

[7] Lavoie P.E., Wilson A.J., Tuffanelli D.L. (1986). Acrodermatitis chronica atrophicans with antecedent lyme disease in a Californian. Case report. Zentralbl Bakteriol Mikrobiol Hyg A.

[8] Kaufman L.D., Gruber B.L., Phillips M.E. (1989). Late cutaneous Lyme disease: acrodermatitis chronica atrophicans. Am J Med.

[9] Edwards L., Hoshaw R.A., Burgdorf W.H. (1992). Acrodermatitis chronica atrophicans. Arch Dermatol.

[10] Scott J.D. (2020). Presentation of acrodermatitis chronica atrophicans rashes on Lyme disease patients in Canada. Healthcare (Basel).

[11] Cardenas-de la Garza J.A., Cuellar-Barboza A., Arvizu-Rivera R.I. (2020). Acrodermatitis chronica atrophicans by Borrelia afzelii in an unusual geographical zone. Rheumatology (Oxford).

[12] Allen M.S., Kilgore R.J., Zhang Y. (2025). Evidence for the long-distance transport of ticks and tick-borne pathogens by human travellers to Texas, USA. J Travel Med.

[13] Ogrinc K., Maraspin V., Lusa L. (2021). Acrodermatitis chronica atrophicans: clinical and microbiological characteristics of a cohort of 693 Slovenian patients. J Intern Med.

[14] Cerar T., Strle F., Stupica D. (2016). Differences in genotype, clinical features, and inflammatory potential of Borrelia burgdorferi sensu stricto strains from Europe and the United States. Emerg Infect Dis.

